# Simultaneous Improvement of Surface Wettability and UV Resistance of Wood with Lignin-Based Treatments

**DOI:** 10.3390/polym15163409

**Published:** 2023-08-15

**Authors:** Rene Herrera, Faksawat Poohphajai, Anna Sandak, Oihana Gordobil

**Affiliations:** 1 Department of Chemical and Environmental Engineering, University of the Basque Country (UPV/EHU), Plaza Europa 1, 20018 Donostia-San Sebastián, Spain; 2 InnoRenew CoE, Livade 6a, 6310 Izola, Slovenia; faksawat.poohphajai@innorenew.eu (F.P.); anna.sandak@innorenew.eu (A.S.); 3 Department of Bioproducts and Biosystems, Aalto University School of Chemical Engineering, 00076 Aalto, Finland; 4 Faculty of Mathematics, Natural Sciences and Information Technologies, University of Primorska, Glagoljaška 8, 6000 Koper, Slovenia; 5 Andrej Marušič Institute, University of Primorska, Titov Trg 4, 6000 Koper, Slovenia

**Keywords:** wood modification treatments, lignin treatments, enhanced hydrophobicity, UV protection, moisture content stabilisation

## Abstract

Recent advancements in wood modification aim to enhance the inherent qualities of this versatile biological material, which includes renewability, ease of processing, and thermal insulation. This study focuses on evaluating the effectiveness of lignin as a protective agent for less durable wood species, namely, *Pinus nigra* and *Fagus sylvatica* L. The impregnation of wood with three various forms of lignin, such as kraft lignin, acetylated kraft lignin, and lignin nanoparticles, was carried out using the vacuum technique at room conditions. The results showed that the treatments significantly improve the hydrophobicity of wood surfaces, particularly in pine wood, and provide protection against UV ageing. Additionally, the treatments contributed to the stabilisation of moisture content at different humidity levels. Although slight colour variations were observed, their impact on the visual appearance was minimal, and the thermal analysis confirmed enhanced thermal properties. Additionally, plasma treatment further enhanced hydrophobicity after treatments, offering potential benefits in terms of moisture resistance and durability. The findings of this study highlight the promising effects of lignin-based treatments on wood properties, providing sustainable solutions for wood protection in various sectors. However, further optimisation is needed to fully explore the potential of lignin and lignin nanoparticles.

## 1. Introduction

Wood is a highly adaptable biological material with numerous advantages, including its renewability, ease of processing, thermal, and acoustic insulation properties. In contrast to commonly used fossil-based materials like concrete, steel, and plastics, wood offers additional benefits such as carbon fixation and the ability to reduce carbon footprint [1,2]. However, due to its hydrophilic nature, wood tends to expand or shrink, which can compromise its dimensional stability, durability, and restrict its range of applications. Consequently, the natural characteristics of wood necessitate treatments to address these issues and extend its service life, thereby expanding the areas for potential applications [3].

Furthermore, wood is often perceived as a less durable material, and the visual appearance of wood-based products plays a crucial role, particularly since wood tends to undergo colour variations when exposed to direct sunlight, resulting in a general greying or darkening effect [4,5]. Additionally, excessive exposure to water accelerates the process of photo degradation, as it opens up the cell wall regions of the wood, making them susceptible to ultraviolet (UV) radiation [6]. The energy carried by UV radiation can lead to the degradation of non-structural components within the wood. These natural effects pose significant challenges to the widespread acceptance of wood as a bio-building material, requiring measures to enhance the service life of wood products [7,8].

Currently, various methods are employed to protect wood from external factors, including chemical modifications, thermal treatments, and the application of additives or chemicals as coatings or penetrating finishes with protective formulations [9,10,11,12]. These approaches often involve the covalent bonding of chemicals, altering wood moisture sorption properties or filling the cell wall with chemical compounds to block hygroscopic groups and reduce sorption sites. These methods contribute to the long-term durability and enhance the dimensional stability of wood. To address concerns related to the toxicity and environmental impact of the chemical treatments traditionally used, there remains a need for sustainable and bio-based solutions that target multifunctional material protection while addressing the limitations of current approaches.

Innovative wood treatments explore the use of natural bioactive additives or products that can be impregnated into the wood, as well as the use of micro or nanocarriers that can deliver components at various scales, targeting multiple properties [13,14]. In this context, lignin, a macromolecule abundant in nature, emerges as one of the most promising raw materials. This polyphenolic polymer is primarily derived from the underutilised byproduct of the pulp and paper industry (as precipitated kraft pulping black liquor), but is also a native and intrinsic component of wood [15].

The utilisation of lignin for wood protection presents an interesting alternative, given its significant phenolic content and inherent functionalities, such as pathogen resistance, thermal stability, biodegradability, antioxidant activity, and UV radiation absorption [15,16,17]. However, to unlock the full potential of lignin in wood applications, modifications are necessary to break down its molecular structure and enhance its reactivity. Gordobil et al. 2016 [18] used esterified organosolv lignins isolated from hardwood and softwood as protective agent for wood products resulting in a stable hydrophobic and oleophobic behaviour on wood veneers over time, which was confirmed by the accelerated aging test. Other authors [19] evaluated the use of lignin nanoparticles for wood surface treatment using a dip-coating technique and observed higher protection to UV irradiation and oxidation of treated than untreated wood samples. Additionally, an innovative approach to wood treatment involves surface preparation through plasma treatment, which offers an environmentally friendly method to modify or enhance the effects of applied products on wood surfaces [20].

In this study, the potential use of lignin as a sustainable protective agent for less durable wood species, such as beech and pine, was evaluated. The objective was to simultaneously improve relevant properties of wood, such as dimensional stability and UV resistance, by means of sustainable and eco-friendly modification process to enhance its service life performance. Simple vacuum impregnation methodologies were employed to impregnate lignin in various forms, and their effectiveness in reducing the hydrophilic nature of wood and protecting its surface against UV ageing was compared. To enhance wood hydrophobicity, kraft lignin was acetylated to shield the phenolic hydroxyl groups, and lignin nanoparticles were utilised to address their inherent heterogeneity, making them more homogeneous, and thus to improve stability. The results were evaluated based on the targeted properties, and the hydrophobic effect was further enhanced through the application of plasma treatment.

## 2. Materials and Methods

### 2.1. Wood Material

Samples from Slovenian plantation-grown wood were used for this study, the softwood species European black pine (*Pinus nigra*) and the hardwood species European beech (*Fagus sylvatica* L.). Samples of heartwood boards free of defects were cut with dimensions of 2 mm (rad.) × 15 mm (tang.) × 30 mm (long.) and conditioned (moisture content: 6.75% (*Pinus nigra*); 7.83% (*Fagus sylvatica* L.), both at 25 °C; 65% relative humidity). Thirty replicates were used for each modification set and for the reference set. 

### 2.2. Lignin Material

Three different types of lignin were used to impregnate the wood: (1) Softwood kraft lignin (L) isolated from the Lignoboost process [21], (2) softwood kraft acetylated lignin (AL) [22], and (3) softwood kraft lignin nanoparticles (LNPs) produced in a pilot plant with an average size of 183.6 nm [23]. For the impregnation process, L and AL were prepared in NaOH solution (1%) at a concentration of 2% (*w*/*v*) and LNPs in water at a concentration of 2% wt.

### 2.3. Impregnation Methodology

Each set of samples was kept at 50 °C for 48 h and then weighed and impregnated with lignin solutions in a vessel connected to a pump, applying a vacuum impregnation cycle for approximately 2 h at room temperature. After the impregnation process, the excess product was removed by rinsing wood with water, and the samples were conditioned at 50 °C for 48 h. 

### 2.4. Samples Characterisation

#### 2.4.1. Physical Properties

The physical changes in wood after impregnations were measured in terms of weight percent gain (*WPG*), absorption dose (*AD*), and density (ρ). These parameters were calculated using the following Equations (1–3):

(1) $$WPG\left(\%\right)=\frac{W_{2}-W_{1}}{W_{1}}\times 100$$

(2) $$AD\left(\%\right)=\frac{W_{3}-W_{1}}{W_{1}}\times 100$$

(3) $$\rho \left(kg/m^{3}\right)=\frac{W_{3}}{V_{3}}$$

where *w*_1_ is the oven dry weight of the sample (50 °C) before impregnation (*g*); *w*_2_ is the oven dry weight of sample (50 °C) after impregnation (*g*); *w*_3_ is the wet weight of sample (23 °C, 65% RH); and *v*_3_ is the volume of sample (23 °C, 65% RH).

#### 2.4.2. Wettability and Surface Free Energy

Changes in wettability and surface free energy of wood were evaluated by the sessile-drop technique at the state of equilibrium contact angle θ using optical tensiometer Attention Theta Flex Auto 4 (Biolin Scientific, Gothenburg, Sweden). Three replica measurements were performed on each sample (10 samples per treatment) with distilled water, ethylene glycol, and diiodomethane as test liquids. The measurement of the drop shape (volume 4 μL) started at the initial drop contact with the assessed sample surface and lasted for 60 s. The free surface energy was calculated from the interactions of the liquid and the solid states following the routine proposed by Owens, Wendt, Rabel and Kaelble (OWRK). The total surface free energy (*γtot*), as well as its polar (*γp*) and disperse (*γd*) components were determined for all samples. A multiple comparison procedure analysis of variance (ANOVA) was used to determine which means were significantly different from others, as well as the confidence levels and Tukey significant difference (TSD) was applied after rejecting the null hypothesis.

#### 2.4.3. Dimensional Stability

For each set of treatments, water sorption and desorption isotherms were calculated using a dynamic vapour sorption apparatus (DVS—surface measurement system). Each sample was cut and a small piece (±20 mg) was taken including the surface and the internal part of the wood. It was then placed on an aluminium plate connected to an ultra-sensitive microbalance capable of recording mass changes at a resolution of 0.1 mg at established sorption–desorption conditions. Climate control is assured by mixing dry nitrogen (0% RH) with saturated water vapour (100% RH). The mixture of both gases was controlled in the closed loop system by continuous monitoring of the relative humidity with ultrasonic (time of flight) sensors. The samples were subjected to a gradual increase in relative humidity (20, 40, 60, 80, and 95% RH), followed by a sequential reduction to 0% RH. The instrument maintained the sample at a constant RH until the weight change per minute fell below 0.002% (dm/dt = 0.002) for at least 15 min. The maximum period of the sorption–desorption step was set as 360 min. The moisture content was computed on the dry mass basis (absolute moisture content) as a percentage ratio of the water mass to the dry matter’s mass of the tested material. From the obtained data, the hysteresis and the sorption–desorption curves after two consecutive cycles were calculated, which is a recommended protocol for all materials of unknown sorption characteristics. These may be altered by the permanent physical–chemical changes (or chemical reactions) occurring in the presence (or absence) of moisture.

#### 2.4.4. UV Stability and Colour Changes

A set of samples (5 samples per treatment) was subjected to cycles of UVA (100%, 8 mW/cm^2^), UVB (100%, 2.4 mW/cm^2^), and UVC (20%, 10 mW/cm^2^) radiation (Irradiation chamber Opsytec) at 15 cm and 25 °C. The effect of radiation was monitored by measuring the colour changes after 300 h of UV cycles. Samples were scanned with an office scanner HP Scanjet 2710 (300 dpi, 24 bit) and saved as TIF files. Colour changes were assessed by means of a MicroFlash 200D spectrophotometer (DataColor Int, Lawrenceville, IL, USA) following the CIE Lab system where colour is expressed with three parameters: L* (lightness), a* (red-green tone), and b* (yellow-blue tone). The selected illuminant was D65 and the viewer angle was 10°. Five replica measurements were performed on each sample and the confidence levels and TSD were applied in the same way as was described in Section 2.4.2. 

#### 2.4.5. Thermal Stability

Thermogravimetric analyses were performed using a thermogravimetric analyser Discovery TGA-5500 (Waters TA Instruments, New Castle, DE, USA). For the thermal analysis, 5–10 mg of each wood sample was cut including the surface and the internal part of the wood, then placed in a platinum crucible and analysed under N_2_ and O_2_ atmosphere (25 mL/min) from 25 to 800 °C with a heating rate of 20 °C/min. Thermogravimetric (TG) and derivative thermogravimetric (DTG) data generated by the instruments were decoded using TA Instruments TRIOS software (2021).

### 2.5. Product Performance

#### 2.5.1. Measurement of Leachability and Performance

Leaching tests were performed according to EN84 with some modifications. Briefly, each set of treatments (5 samples each) was subjected to vacuum for 2 h and then immersed into 500 mL of distilled water for 240 h, with the water being changed every 24 h. Subsequently, the wood samples were collected and dried at 50 °C for 48 h to calculate the weight loss, wettability changes by WCA measurements, and colour changes.

#### 2.5.2. Surface Post Treatment by Plasma Technology

An atmospheric plasma diffuse coplanar surface barrier discharge (DCSBD) was used as a post treatment method with the objective of determining changes in the hydrophobicity of the treated surfaces. Five samples from each treatment were exposed to a micro discharge for 2 s/mm of sample at 1 mm distance from the surface under ambient conditions. After plasma exposure, samples were conditioned (23 °C, 65% RH) and the WCA was measured according to the procedure described in Section 2.4.2.

## 3. Results and Discussion

### 3.1. Physical Properties

The degree of impregnation was evaluated through the weight percent gain (*WPG*) resulting from the treatments applied to the wood, and by calculating the absorption dose of the product. The absorption dose is the difference between the oven-dried weight and the conditioned weight of the samples (Table 1). The results showed no statistical differences in *WPG* among the different treatments. However, when considering the wood species, it was observed that hardwood samples (beech) exhibited *WPG* values that were 40% lower compared to softwood samples (pine), irrespective of the specific lignin treatment employed. Additionally, the absorption dose of the products was similar in both species, with a slightly higher percentage observed in the softwood samples. The results suggest that beech, which has a higher density and cell wall thickness compared to softwood, exhibits reduced permeability and slower moisture loss during the conditioning process. This is supported by the slight increase in moisture content (MC) observed after treatment.

Nevertheless, a linear correlation was identified between *WPG* and density for both pine wood (R^2^ = 0.996) and beech wood (R^2^ = 0.981). The findings suggest that the effectiveness of the lignin treatment is primarily influenced by the substrate. In general, the treatments are better suited for low-density species [24] since they possess a higher impregnation capacity and have a more suitable anatomical configuration [25,26]. In contrast, denser species may require longer impregnation times or cycles to penetrate the cell wall adequately, thereby achieving more optimal results.

### 3.2. Wetting Behaviour and Hygroscopic Properties

**Figure 1:**
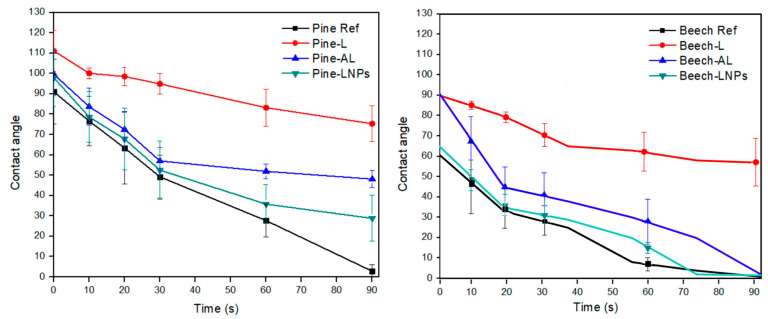
WCA of impregnated wood samples over time.

To assess the effectiveness of water repellence, the static water contact angle (WCA) was evaluated over time (Figure 1) and changes in the surface free energy of the wood were calculated (Table 2). Both treated wood species exhibited an increase in WCA, but higher values were observed in the softwood species, showing hydrophobic values (WCA > 90°), during the first few seconds in all treatments except for beech-LNPs treatment, which was slightly higher than the reference hardwood sample. After 90 s, the WCA were smaller in all samples, but the values for pine-L and beech-L treatments remained relatively steady, indicating that this treatment allows for only a partial wetting of wood surfaces. It is important to note that all samples were sanded (280-grit sandpaper) to achieve the same surface roughness, ensuring that the evaluation of surface properties occurred under consistent conditions [27]. Consequently, the variations observed in WCA were primarily attributed to the surface chemistry of each wood species and their interaction with the functional groups present in the lignin solutions (mainly carboxyl, methoxy, and hydroxyl groups) [28].

The analysis of the surface free energy components of wood showed that the polar share was reduced to values below 1 mJ/m^2^ in the treatments applied to pine wood and beech-AL (Table 2). This indicates a decrease in the wettability of the wood after these treatments, particularly with acetylated lignin, where higher water contact angles (WCA) and lower polar interaction on the surfaces were observed. This shift toward lower wettability in pine wood treatments and beech-AL can be attributed to the modification of the surface free energy as a consequence of the applied treatments. Acetylated lignin, for example, may introduce hydrophobic characteristics to the wood surface, reducing the polar interaction and making it less susceptible to wetting [29].

**Figure 2:**
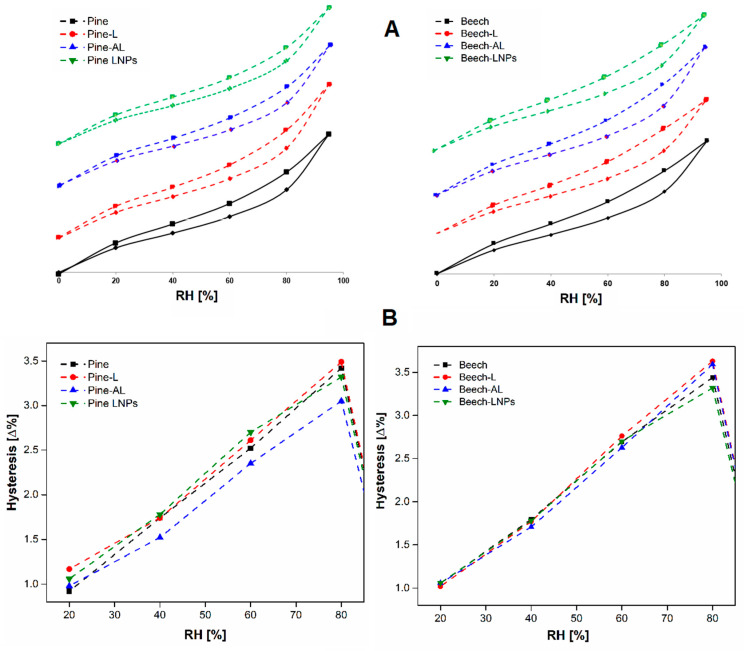
Sorption isotherms (**A**) and hysteresis (**B**) plotted as a function of relative humidity for reference and treated wood specimens measured by dynamic vapour sorption (DVS).

To study the changes in the dimensional stability of wood samples at different moisture contents, experimental adsorption and desorption tests were performed and the sorption isotherms and the hysteresis behaviour were analysed. Figure 2a shows similar isotherms of both untreated and lignin-based treated wood, with a sigmoid shape isotherm (type II), typical adsorption performance of monolayer-multilayer lignocellulosic materials [30,31].

Analysis of the hysteresis plot (Figure 2b) revealed varying effects on the hygroscopic behaviour of wood among the treatments and wood species. The width of the hysteresis loop in wood depends on internal bonding between individual cell wall polymers. With the increased number of bonds, the loop increases [32]. In the case of treatments on softwood, pine-AL exhibited a stable performance across the entire moisture range, while pine-LNPs showed stability from 75% relative humidity (RH) onwards. On the other hand, in hardwood treatments, beech-AL displayed minor differences in equilibrium moisture content (EMC) within the 35–65% RH range, while beech-LNPs showed similar behaviour from 65% RH. However, both pine-L and beech-L did not demonstrate significant improvements in dimensional stability, exhibiting similar dimensional changes to those observed in the untreated samples.

Furthermore, the reduced effect of LNPs treatment on hygroscopic properties could be explained by the mechanism of lignin nanoparticle formation, in which dissolved lignin is precipitated in water (antisolvent). This process leads to the formation of a core-shell structure, where hydrophobic regions are assembled first to form the particle’s core, while the most polar molecules are adsorbed on the surface, creating a layer with low water repellence.

The observed differences in moisture sorption and release rates can be attributed to various factors, including the wood microstructure, chemical composition, and pore structure [3]. These characteristics play a significant role in determining the dimensional stability of wood. In this study, it was evident that the softwood species, with their distinct microstructure and chemical composition, may possess favourable attributes that contribute to their enhanced dimensional stability when compared to hardwood. Moreover, the changes in the EMC after treatments are crucial in determining a wood’s ability to withstand dimensional changes. The treatments had a notable impact on the EMC, particularly in the case of beech wood. This suggests the need for potential improvements in impregnation parameters or sample preparation to enhance its dimensional stability. Although the dynamic vapour sorption (DVS) analysis does not follow the standard for the determination of moisture stability on wood samples, it is considered a useful technique for the determination of sorption isotherms of materials providing numerous advantages in comparison with a traditional static method [33,34].

### 3.3. Colour Changes and UV Stability

**Figure 3:**
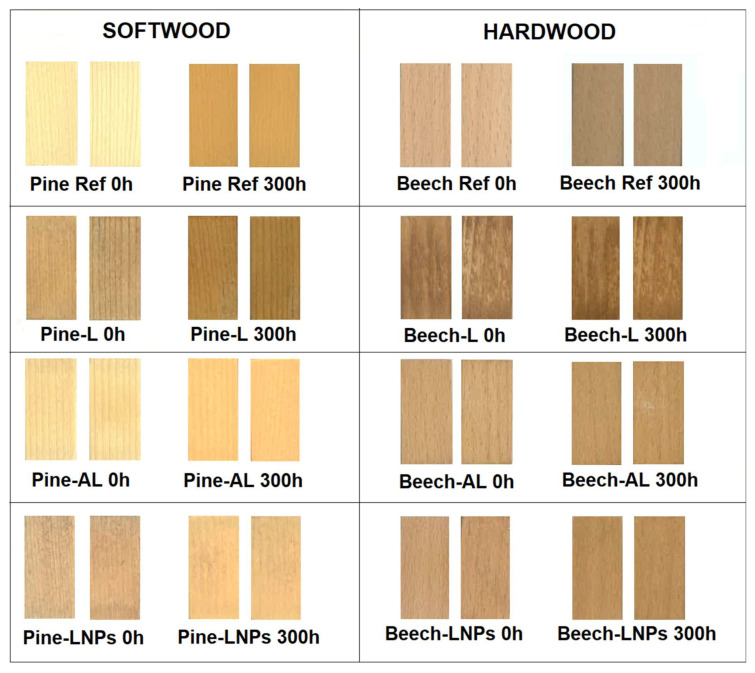
Sample scans before and after 300 h of UV radiation.

The colour changes observed in the treated samples compared to the reference samples indicate the influence of the treatment methods on the visual appearance of the wood. As observed in Figure 3, the samples underwent a change in their original colour after treatment, exhibiting lower lightness values (L*) compared to the reference samples (Table 3). This effect was particularly prominent in pine-L, pine-LNPs, and beech-L treatments, where the colour appeared darker and less homogeneous compared to other treatments. In contrast, the appearance of pine-AL, beech-AL, and beech-LNPs treatments was similar to the reference samples, with minor changes in the colour parameters, suggesting a relatively minor impact on the visual appearance. The colour of treated wood might be later optimised by changing the source of lignin used for impregnation [19].

Furthermore, the results from the UV ageing tests revealed interesting findings regarding colour stability. After 300 h of UV exposure, the total colour difference (Δcolour) was higher in the reference samples compared to the treated samples, which implies that the treated samples exhibited better resistance to colour degradation caused by UV radiation. The specific order of colour stability varied for each species, in which the following variations were observed: Pine-Ref > Pine-AL > Pine-L > Pine-LNPs, and Beech-Ref > Beech-LNPs > Beech-AL > Beech-L. The colour stability of the treated samples after UV testing can be attributed to the effect of lignin addition. Previous studies have suggested that lignin can act as a natural UV absorber, providing protection against the detrimental effects of UV radiation on wood surfaces [19,35].

### 3.4. Thermal Properties

**Figure 4:**
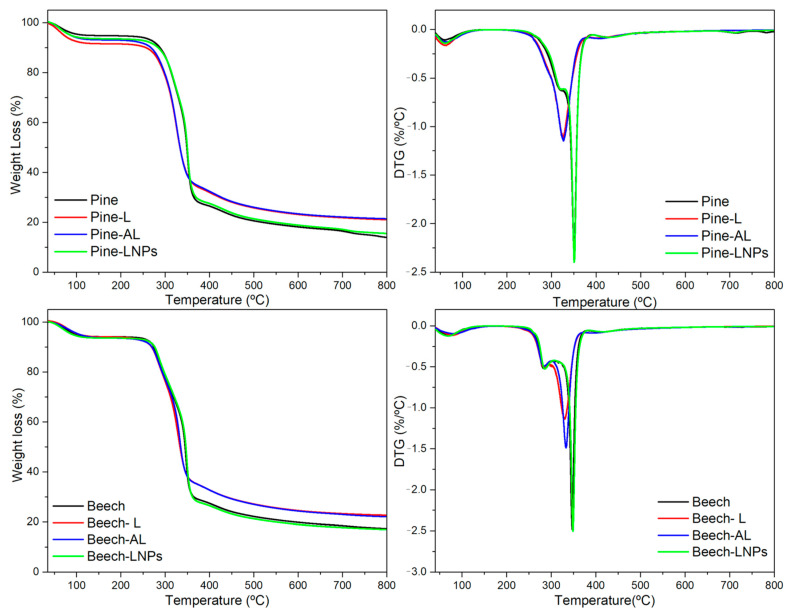
TGA and DTG of treated samples and references in N_2_.

Thermogravimetric analyses were performed under inert and oxidative atmospheres to assess the decomposition behaviour and thermal characteristics of wood following impregnation. Figure 4 displays the TG profiles in N_2_ and the corresponding derivative curves, revealing distinct degradation temperatures between the reference samples (pine and beech) and the samples treated with lignin and acetylated lignin. In the case of pine wood, the treatments exhibited a less prominent peak at approximately 330 °C, while the reference and LNPs-treated samples displayed a shoulder at this temperature and a more pronounced peak at around 349 °C. Similarly, for beech wood, thermal degradation occurred in two distinct stages, featuring a common, less intense peak at 265–275 °C and a subsequent peak representing maximum weight loss rates at approximately 330 °C for beech-L and beech-AL, and around 345 °C for beech-ref and beech-LNPs. Remarkably, the results indicated that impregnation with lignin and acetylated lignin may have contributed to the formation of more stable compounds during the thermal decomposition process, resulting in a lower mass loss with higher residual mass (>20%) in these treatments.

Similar trends were observed in the TGA analysis conducted under oxidative atmosphere (O_2_), where lower degradation peaks were observed (Table 4). Notably, in the case of beech samples (all treatments), the degradation temperature (T_10_) was similar to the reference sample but with a reduced mass loss, indicating an oxidative stability and reduced flammability [36]. The observed reduction in mass loss at the degradation steps further suggests enhanced thermal stability in both species treated with lignin and acetylated lignin.

The results obtained from the TGA analyses provide valuable insights into the thermal performance and stability of wood samples, especially after impregnation with lignin and acetylated lignin. The lower degradation peaks observed in both N_2_ and O_2_ atmospheres suggest that the impregnation process influenced the decomposition behaviour of the wood components. This can be attributed to the interaction between lignin or acetylated lignin and the wood matrix, leading to the formation of a protective layer or barrier that inhibits the access of oxygen to the wood surface, thereby reducing the susceptibility to oxidative degradation and enhancing the overall fire resistance properties [37].

### 3.5. Product Performance: Properties after Leaching Cycle

The retention levels, WCA, and colour changes in the treated wood after a leaching cycle are presented in Table 5. The *WPG* loss in all treated samples was lower than the initial *WPG*, particularly in softwood, where more than 70% of the product was retained in all treated samples. Conversely, the retention of lignin in hardwood samples was negligible for beech-L (1%), while it reached a maximum of 58% for beech-LNPs. The observed *WPG* loss in all cases can be attributed primarily to the removal of water-soluble wood extracts and unreacted solution. The effectiveness of the impregnation process is directly correlated with the permeability of the wood species. It can be concluded that pine wood is suitable for impregnation with lignin-based treatments, as it exhibits lower leaching compared to beech wood. Treatment of beech was found to be less effective, most likely due to a more closed cell structure, making it less receptive to lignin impregnation.

The type of lignin affects treatment effectiveness. It was observed that impregnation with unmodified lignin was more leachable in both species. This indicates that it is more difficult for untreated kraft lignin to penetrate the wood structure, and thus further modifications to the lignin or adjustments in particle size are necessary to enhance its retention [38]. The water contact angle (WCA) was measured after the leaching test to assess changes in the surface wettability of the treated samples. It is noteworthy that all treated samples exhibited similar tendencies in terms of WCA values after 60 s. However, it is particularly interesting to notice the hydrophobic behaviour in the pine-L treatment. This suggests that the impregnation of pine wood with the specific treatment resulted in a surface that repels water, indicating improved water resistance and potential durability of the treated wood.

Additionally, colour changes (∆L, ∆a, ∆b, ∆E) were measured after leaching to assess the impact on the appearance, considering that the original wood colour was noticeably altered due to the treatment (Figure 2). The results showed no tendency regarding the treatment or wood species after leaching. However, it is noteworthy that both the pine-L treatment and the beech-LNPs treatment overall exhibited reduced colour changes (∆colour). Furthermore, it was observed that pine-LNPs and beech-AL treatments shift toward a darker surface and reddish tone on the surfaces, as indicated by their negative lightness values (∆L) and positive ∆a values.

### 3.6. Improvement in Water Repellence: Plasma Treatment on Surface

**Figure 5:**
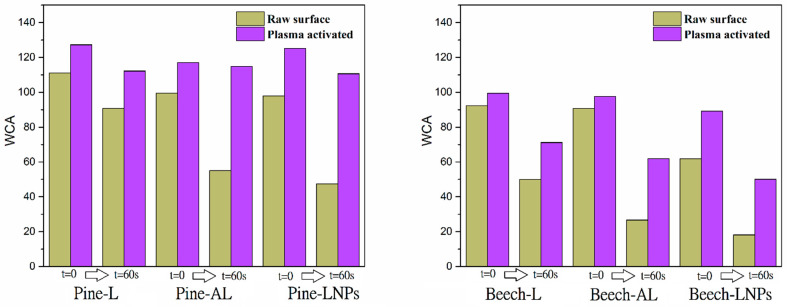
Changes in WCA of impregnated wood samples at t = 0 and after 60 s.

All target properties were enhanced after the lignin-based treatments. However, to explore potential post treatments that could enhance the effectiveness of lignin treatments, samples were subjected to micro discharge using ambient air as the process gas with an atmospheric plasma (DCSBD). First, reference samples were tested, resulting in a decrease of approximately 30% in WCA from its initial value for pine wood and approximately 50% for beech wood. This effect indicates that surface activation in the reference samples led to increased liquid absorption rather than repellence, aligning with findings reported by other researchers. [39]. With regards to the treated samples, the results were compared with the obtained values of water contact angle (WCA) without plasma treatment (Figure 5). All the treated samples, after plasma micro discharge, showed a decrease in polarity at the initial time (WCA > 10%). This tendency remained constant over time, with improved WCA values exceeding 50%. The treated surfaces exhibited hydrophobic characteristics (WCAs > 90°), particularly in the case of softwood surfaces (pine). This hydrophobic nature was maintained over time, with contact angles exceeding 110° after 60 s.

The resulting values after surface plasma treatment suggest that the changes in surface chemistry or composition, such as oxidation or modification of hydroxyl, carbonyl, and carboxyl groups associated with the added lignin, could be responsible for the observed effects. In previous studies using DCSBD treatment to modify surface polarity, researchers reported an increase in water contact angles (WCA) due to the degradation of hemicellulose on the surface during the discharge process [40,41]. These findings indicate that plasma treatment has the potential to further alter the surface properties of the samples and enhance their hydrophobicity.

However, it is important to note that the exact mechanisms underlying these changes in surface structure and chemistry are still not fully understood. Further research is needed to investigate the specific molecular interactions and transformations that occur during plasma treatment. Additionally, the long-term stability and durability of the modified surfaces should be assessed to determine their practical implementation in various applications.

## 4. Conclusions

The results of this study provide valuable insights into the effects of kraft lignin (L), acetylated lignin (AL), and lignin nanoparticles (LNPs) treatment on wood properties when applied at ambient temperature within short impregnation cycles. Similar weight percent gain (*WPG*) was found among the treatments. However, hardwood samples (beech) exhibited lower *WPG* values than softwood samples (pine), indicating reduced permeability and slower moisture loss during the conditioning process. The treatments improved the hydrophobicity of the wood surfaces, with higher water contact angles (WCA), particularly in softwood species. This suggests that the treatments had a more pronounced effect on the surface chemistry of pine wood, allowing for only partial wetting of the surfaces.

The hygroscopic behaviour of the treated wood varied among the different treatments and wood species. While pine-AL and pine-LNPs showed stability in terms of equilibrium moisture content (EMC) at higher relative humidity levels, beech-AL and beech-LNPs exhibited minor differences in EMC within specific RH ranges. Although the treatments resulted in a darker and less homogeneous colour of the samples, pine-AL, beech-AL, and beech-LNPs treatments exhibited relatively minor changes in colour parameters, indicating a lesser impact on the visual appearance.

Thermal analysis showed lower degradation in L and AL treatments, indicating that these treatments contributed to the formation of a protective layer or barrier, reducing susceptibility to oxidative degradation and enhancing fire resistance properties. Moreover, UV ageing tests indicated that the treated samples exhibited better resistance to degradation caused by UV radiation compared to the reference samples. The leaching tests demonstrated the effectiveness of the impregnation process. Pine wood showed less leaching compared to beech wood, suggesting its suitability for lignin-based treatments.

Additionally, the micro discharge plasma treatment (DSCBD) applied to the treated surfaces increased their hydrophobic character with WCA values exceeding 90° and remaining consistent over time. This surface post treatment could improve moisture resistance and durability of lignin-based impregnations.

In summary, the investigated lignin-based treatments showed promising effects on various wood properties, including moisture sorption, hydrophobicity, colour stability, thermal performance, and leaching. Bio-based wood treatments simultaneously improving relevant wood properties are particularly interesting for future industrial upscale due to the lack of sustainable and eco-friendly solutions targeting multifunctional material protection. Conducted research contributes toward understanding the effects of bio-based treatments on different wood species and provides insights for their application in various sectors. Further research is necessary to explore the full potential of lignin and further improve its effectiveness, optimisation, as well as to upscale the treatment process.

## Figures and Tables

**Table 1:** Physical properties of wood after lignin-based treatments.

Specimen	*WPG* [%] *	Absorption Dose [%]	MC (65% RH–25 °C) [%] *	Density [kg/m^3^] *	Increase in Density [%]
Pine Ref	-	-	8.49 (0.37)	437.84 (13.67)	-
Pine-L	2.15 (0.27)	10.38	8.07 (0.32)	450.22 (13.87)	2.14
Pine-AL	2.09 (0.20)	9.80	7.53 (0.24)	444.27 (15.12)	2.09
Pine-LNPs	1.98 (0.28)	9.84	7.80 (0.25)	445.84 (11.84)	1.89
Beech Ref	-	-	6.93 (0.50)	741.21 (28.94)	-
Beech-L	0.81 (0.20)	8.91	8.08 (0.16)	749.72 (28.87)	0.77
Beech-AL	0.74 (0.18)	8.82	8.14 (0.16)	754.04 (27.39)	0.63
Beech-LNPs	1.24 (0.27)	8.63	7.25 (0.41)	739.59(26.91)	1.27

* The difference of the means of each measurement is shown in parenthesis.

**Table 2:** Contact angle of wood with different liquids and changes in surface free energy after lignin-based treatments.

Specimen	WCA [°]	FCA [°]	DCA [°]	Surface Free Energy [mJ/m²]		
				γ_Polar_	γ_disperse_	γ_TOTAL_
Pine Ref	91.0 (6.31) **	25.9 (3.56) *	31.9 (3.89) *	2.5	47.7	50.2
Pine-L	111.1 (5.78) ***	50.3 (4.32) *	11.3 (1.14) **	0.5	57.5	60.0
Pine-AL	99.6 (5.37) ***	49.6 (4.21) *	10.1 (1.12) **	0.3	57.1	57.4
Pine-LNPs	97.9 (5.96) **	26.6 (2.45) *	32.3 (3.78) *	0.8	51.1	51.9
Beech Ref	56.8 (5.56) **	31.7 (2.55) **	17.2 (2.22) *	7.3	44.3	51.6
Beech-L	92.3 (4.06) *	39.9 (3.89) **	12.2 (1.65) *	2.7	48.2	50.9
Beech-AL	90.7 (4.59) *	28.7 (2.66) **	16.3 (2.47) *	0.1	57.6	57.7
Beech-LNPs	61.8 (4.69) **	43.2 (3.88) **	12.8 (1.47) *	4.7	44.7	49.4

WCA = water contact angle; FCA = formamide contact angle; DCA: diiodomethane contact angle. The difference of the means of each measurement is shown in parenthesis. Significance: * indicates *p* < 0.05, ** indicates *p* < 0.01, *** indicates *p* > 0.01.

**Table 3:** Lab colour values before and after 300 h of UV ageing test.

Specimen	Initial Colour			Colour after UV Radiation			Δcolour
	L	a	b	L	a	b	
Pine Ref	82.27 (0.42) ***	4.75 (0.32) **	22.86 (0.29) ***	68.73 (1.89) **	13.07 (0.56) **	39.49 (0.77) ***	23.0
Pine-L	65.89 (1.23) *	9.45 (0.89) ***	27.41 (0.26) ***	60.08 (0.65) *	14.35 (0.28) ***	37.86 (0.49) *	13.0
Pine-AL	79.24 (0.85) ***	5.55 (0.55) **	26.49 (0.31) ***	67.60 (0.33) **	12.08 (0.72) **	37.74 (0.44) *	17.6
Pine-LNPs	66.56 (0.38) *	11.38 (0.76) ***	24.87 (0.21) ***	60.19 (1.25) *	13.57 (0.60) **	34.97 (0.12) ***	12.2
Beech Ref	68.55 (1.02) ***	10.29 (1.18) *	20.52 (0.12) ***	62.52 (1.23) **	12.77 (0.33) *	27.90 (0.42) ***	9.9
Beech-L	54.90 (1.56) ***	13.07 (1.45) **	25.40 (0.66) *	52.63 (0.62) ***	14.62 (0.56) ***	29.93 (0.46) *	5.4
Beech-AL	65.64 (1.12) **	10.20 (0.88) *	25.73 (0.42) *	61.38 (0.29) **	12.17 (0.32) **	29.42 (0.30) *	6.0
Beech-LNPs	63.93 (0.96) **	11.85 (0.94) **	24.42 (0.22) ***	58.90 (0.90) ***	13.09 (0.74) *	29.39 (0.48) *	7.3

The difference of the means of each measurement is shown in parenthesis. Significance: * indicates *p* < 0.05, ** indicates *p* < 0.01, *** indicates *p* > 0.01.

**Table 4:** Parameters determined from the thermogravimetric analysis in O_2_.

Specimen	T_10_ (°C)	T_50_ (°C)	T_max_ (°C)	Residue at 800 °C (%)
Pine Ref	274.7	351.6	352.8	14.3
Pine-L	261.9	325.8	319.7	21.1
Pine-AL	251.4	331.4	325.2	20.9
Pine-LNPs	269.8	353.2	355.1	15.5
Beech Ref	264.9	341.3	344.6	17.3
Beech-L	261.2	336.4	337.9	22.7
Beech-AL	263.7	332.7	335.5	22.0
Beech-LNPs	268.5	343.2	346.2	16.9

T_10_ = temperature at 10% of sample degradation; T_50_ = temperature at 50% of sample degradation; T_max_ = temperature of maximum degradation.

**Table 5:** *WPG* loss and colour changes after leaching.

Specimen	*WPG* Loss after Leaching [%]	Product Retained [%]	WCA after Leaching (t = 60 s)	Colour Changes			
				ΔL	Δa	Δb	Δcolour
Pine Ref	-	-	24.88	0.9	0.0	0.3	0.9
Pine-L	−0.62 (0.27)	71.16	90.80	1.3	0.5	−1.5	2.1
Pine-AL	−0.46 (0.10)	77.99	55.10	6.7	−1.3	−0.9	6.8
Pine-LNPs	−0.47 (0.14)	76.26	47.43	−5.7	2.8	−0.8	5.8
Beech Ref	-	-	10.65	6.7	0.1	−2.2	7.1
Beech-L	−0.80 (0.33)	1.24	49.95	−7.8	2.5	2.3	8.5
Beech-AL	−0.47 (0.09)	36.47	26.65	4.8	−0.3	−1.9	5.2
Beech-LNPs	−0.52 (0.17)	58.06	18.16	0.3	0.9	0.3	1.0

The difference of the means of each measurement is shown in parenthesis.

## Data Availability

The data presented in this study are available on request from the corresponding authors.
